# Computational prediction of the effects of the intra-aortic balloon pump on heart failure with valvular regurgitation using a 3D cardiac electromechanical model

**DOI:** 10.1007/s11517-017-1731-x

**Published:** 2017-10-23

**Authors:** Chang-Hyun Kim, Kwang-Soup Song, Natalia A. Trayanova, Ki Moo Lim

**Affiliations:** 1 Department of IT Convergence Engineering, Kumoh National Institute of Technology, 61 Daehak-ro, Gumi, Gyeongbuk 39253 Republic of Korea; 2 Department of Biomedical Engineering, Johns Hopkins University, Baltimore, MD USA

**Keywords:** Intra-aortic balloon pump, Aortic regurgitation, Mitral regurgitation, 3D electromechanical model, Stroke volume, Ventricular workload

## Abstract

Intra-aortic balloon pump (IABP) is normally contraindicated in significant aortic regurgitation (AR). It causes and aggravates pre-existing AR while performing well in the event of mitral regurgitation (MR). Indirect parameters, such as the mean systolic pressure, product of heart rate and peak systolic pressure, and pressure–volume are used to quantify the effect of IABP on ventricular workload. However, to date, no studies have directly quantified the reduction in workload with IABP. The goal of this study is to examine the effect of IABP therapy on ventricular mechanics under valvular insufficiency by using a computational model of the heart. For this purpose, the 3D electromechanical model of the failing ventricles used in previous studies was coupled with a lumped parameter model of valvular regurgitation and the IABP-treated vascular system. The IABP therapy was disturbed in terms of reducing the myocardial tension generation and contractile ATP consumption by valvular regurgitation, particularly in the AR condition. The IABP worsened the problem of ventricular expansion induced as a result of the regurgitated blood volume during the diastole under the AR condition. The IABP reduced the LV stroke work in the AR, MR, and no regurgitation conditions. Therefore, the IABP helped the ventricle to pump blood and reduced the ventricular workload. In conclusion, the IABP partially performed its role in the MR condition. However, it was disturbed by the AR and worsened the cardiovascular responses that followed the AR. Therefore, this study computationally proved the reason for the clinical contraindication of IABP in AR patients.

## Introduction

An intra-aortic balloon pump (IABP) is used to increase myocardial oxygen perfusion, while simultaneously increasing cardiac output and decreasing the workload of the ventricle. This is realized via counterpulsation of the IABP. Extant research indicates that valve regurgitation, i.e., backward flow in the heart when a cardiac valve does not close completely, has a significant effect on the cardiac function [1]. Heart valves are located between the atria and the ventricles (the mitral and tricuspid valves) and between the ventricles and the aortas (the aortic and pulmonary aortic valves). The most common heart valve diseases include aortic and mitral insufficiencies. Cardiac responses such as cardiac output and blood pressure vary according to the type of valve that is affected and the severity of the regurgitation. This can also affect the efficacy of the IABP function when patients are treated with IABP therapy [2]. IABP is normally contraindicated in significant aortic regurgitation (AR). It causes and aggravates pre-existing AR while performing well in the event of mitral regurgitation (MR) [3]. Nevertheless, a question of clinical significance arises since the AR can co-exist with the IABP.

Indirect parameters such as the mean systolic pressure, product of heart rate and peak systolic pressure, and pressure–volume are used to quantify the effect of IABP on ventricular workload. However, to date, no studies have quantified the reduction in workload with IABP directly since experimental methods for documenting and evaluating myocardial energy consumption throughout the ventricular volume are hampered by low spatiotemporal resolution. Computational modeling is an alternative approach to overcome this limitation. Previously, a computational model of IABP support was developed using a 3D electromechanical model of failing ventricles in conjunction with a lumped model of the circulatory system [4, 5]. Furthermore, a 3D electromechanical model of failing ventricles with mitral and aortic valve regurgitations was also developed [6]. These models enabled the quantification of changes in the local contractile energy consumption of the myocardium. In this study, IABP function was incorporated into the electromechanical model of a ventricle with mitral and aortic valve regurgitation with a lumped model of the circulatory system.

The goal of this study involves examining the effect of IABP therapy on ventricular mechanics under the valvular insufficiency condition, which includes the AR and MR conditions in the failing ventricle, by using a computational model of the heart. For this purpose, the 3D electromechanical model of the failing ventricles used in previous studies was coupled with a lumped parameter model of valvular regurgitation and the IABP-treated vascular system.

## Methods

### Ventricular electromechanical model

**Fig. 1:**
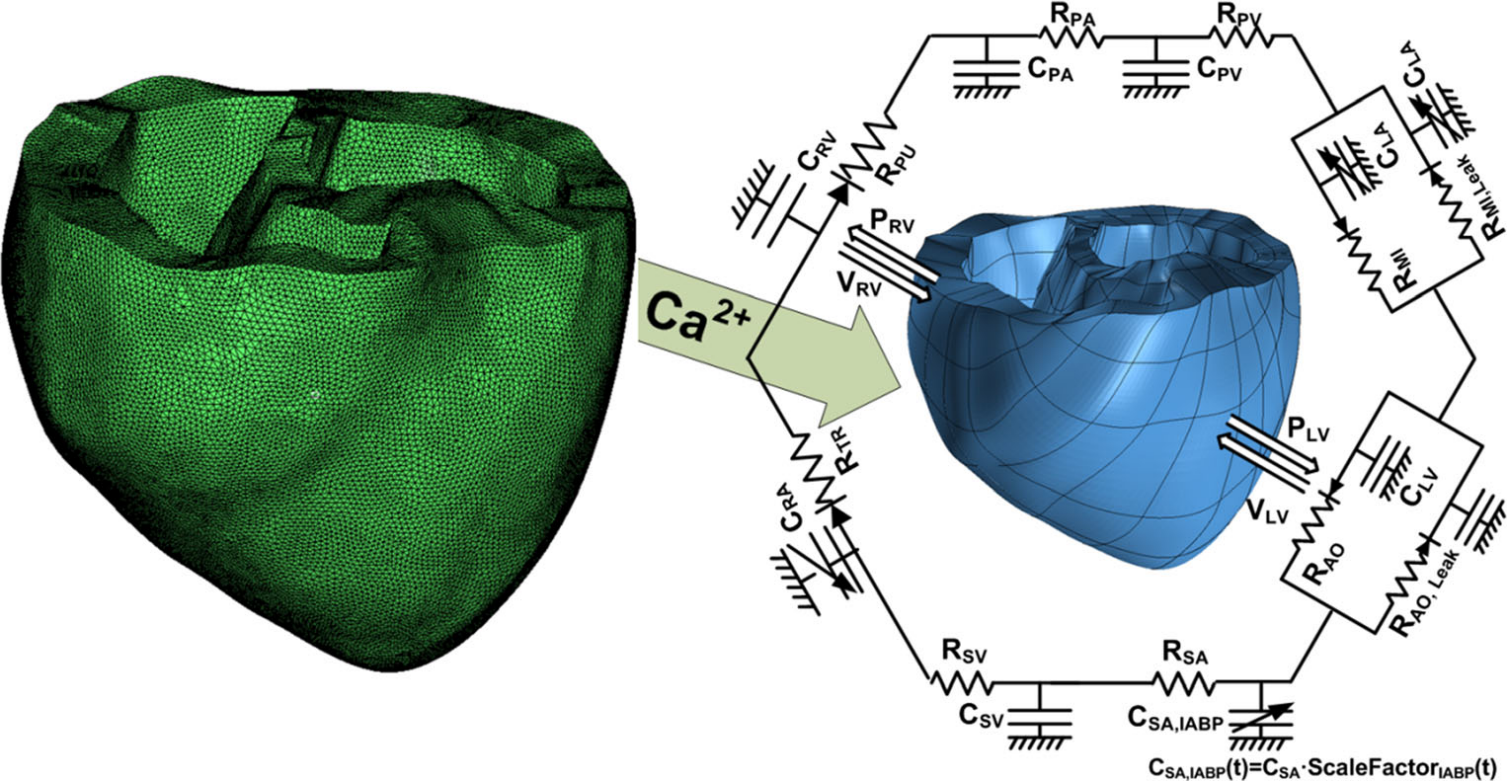
A schematic diagram of the finite element ventricular electromechanical model coupled with the model of IABP-implanted circulatory system with valvular insufficiency. In the figure, *P*_*RV*_ denotes right ventricular pressure, *V*_*RV*_ denotes right ventricular volume, *P*_*LV*_ denotes left ventricular pressure, *V*_*LV*_ denotes left ventricular volume, *R*_*PA*_ denotes pulmonary artery resistance, *C*_*PA*_ denotes pulmonary artery compliance, *R*_*PV*_ denotes pulmonary vein resistance, *C*_*PV*_ denotes pulmonary vein compliance, *R*_*MI*_ denotes mitral valve resistance, *C*_*LA*_ denotes left atrium compliance, *R*_*AO*_ denotes aortic valve resistance, *R*_*SA*_ denotes systemic artery resistance, *C*_*SA,IABP*_ denotes IABP-implanted systemic artery compliance, *R*_*SV*_ denotes systemic vein resistance, *C*_*SV*_ denotes systemic vein compliance, *R*_*TR*_ denotes tricuspid valve resistance, *C*_*RA*_ denotes right atrium compliance, and *R*_*PU*_ denotes pulmonary valve resistance

An MRI-based electromechanical model of the failing canine ventricles developed by previous studies was used to achieve the goals of this study [7]. The ventricular geometry and fiber and laminar sheet architecture of the model were constructed from high-resolution MRI and diffusion tensor MRI scans of canine ventricles involved in heart failure (HF). The model consisted of coupled electrical and mechanical components and a lumped parameter representation of the circulatory system. A schematic diagram of the model is shown in Fig. 1.

The electromechanical model had two dynamic components, namely electrical and mechanical components, as described in a previous study [8]. The electrical component of the model simulated the propagation of transmembrane potential waves by solving monodomain equations on the finite element mesh, which was comprised of 241,725 nodes and 1,298,751 elements in terms of a tetrahedron linear interpolation. The equation described the current flow through ventricular cells that were connected by means of high conductance gap junctions. This electrical property of the gap junction allowed for a continuum representation of current flow in the heart. The 2D Purkinje network proposed by Berenfeld and Jalife [9] was then mapped onto the 3D endocardial surface. Electrical wave propagation through the Purkinje fiber was implemented by solving a 1D wave equation instead of incorporating the ionic model of the Purkinje cell. The current flow in the tissue was driven by the ion exchange across cellular membranes. These processes were represented by the human iconic model proposed by ten Tusscher et al. [10], which represents the current flow through ion channels, pumps, and exchangers in myocyte membranes as well as the subcellular Ca cycling between the cytosol and sarcoplasmic reticulum (SR). The electrical wave propagation in the heart was represented by simultaneously solving the partial differential equation (PDE) for the passive electrical conduction model and the set of ordinary differential equations (ODEs) for the active ionic model. From the electrical simulation results, the spatial distribution of Ca^2+^ transient used as the triggers for the cardiac mechanical model was obtained.

The mechanical component of the model simulated the ventricular contraction. The ventricular contraction was a result of the active tension generated by the myofilaments of the ventricular cells. Ventricular deformation was represented by the equations of passive cardiac mechanics, with the myocardium as an orthotropic (due to fiber and laminar sheet organization), hyperelastic, and nearly incompressible material with passive mechanical properties defined by an exponential strain energy function. The model comprised of 356 nodes and 172 elements in terms of the Hermite polynomial interpolation. The simulation of ventricular contraction was constituted by simultaneously solving the active myofilament model equations along with the equations representing passive cardiac mechanics on the finite element mechanical mesh. The electromechanical model incorporated the biophysical representation of cardiac myofilament dynamics proposed by Rice et al. [11], which represents an excitation–contraction coupling mechanism (cross-bridge cycling induced by Ca^2+^ release). A set of ordinary differential equations and algebraic equations described the binding of Ca^2+^ to troponin C, cooperativity between regulatory proteins, and cross-bridge cycling. A lumped parameter model of the systemic and pulmonic circulatory systems based on a model proposed by Kerckhoffs et al. [12] imposed conditions on ventricular volumes and pressure.

The parameters in the electromechanical model were changed in order to implement the remodeling of electromechanical properties associated with heart failure such that the previous experimental observations [13–15] were matched. Electrical conductivities were reduced by 30% [13], whereas the passive scaling constant of the strain energy function was increased to five times that of the normal value to represent the increased stiffness of the failing myocardium [14]. The peak calcium concentration in the failing cardiomyocyte was reduced to 70% of the value in healthy myocytes to incorporate systolic dysfunction [15]. A similar approach was used in recent studies [4–6].

### Model of IABP function

The systemic and pulmonic circulatory system model is in accordance with that of Kerckhoffs et al., which is expressed by the following mathematical equations [12]:

1 $$ -{R}_{SA}{\dot{Q}}_{SA}+\frac{1}{C_{SA}}{Q}_{SA}={V}_{SV} $$

2 $$ -{R}_{SV}{\dot{Q}}_{SV}+\frac{1}{C_{SV}}{Q}_{SV}={V}_{RA} $$

3 $$ -{R}_{RA}{\dot{Q}}_{RA}+\frac{1}{C_{RA}}{Q}_{RA}={V}_{RV} $$

4 $$ -{R}_{RV}{\dot{Q}}_{RV}+\frac{1}{C_{RV}}{Q}_{RV}={V}_{PA} $$

5 $$ -{R}_{PA}{\dot{Q}}_{PA}+\frac{1}{C_{PA}}{Q}_{PA}={V}_{PV} $$

6 $$ -{R}_{PV}{\dot{Q}}_{PV}+\frac{1}{C_{PV}}{Q}_{PV}={V}_{LA} $$

7 $$ -{R}_{LA}{\dot{Q}}_{LA}+\frac{1}{C_{LA}}{Q}_{LA}={V}_{LV} $$

8 $$ -{R}_{LV}{\dot{Q}}_{LV}+\frac{1}{C_{LV}}{Q}_{LV}={V}_{SA} $$

where *R* is resistance, *Q* is flux, *V* is volume, *C* is compliance, *SA* is systemic artery, *SV* is systemic vein, *RA* is right atrium, *RV* is right ventricle, *PA* is pulmonary artery, *PV* is pulmonary vein, *LA* is left atrium, and *LV* is left ventricle. The subscript symbols represent the compartments of the circulatory system. The list of initial values of these parameters is presented in the Table 1.

**Table 1:** Parameters of the lumped circulatory systems

Variable	Meaning	Value	Unit
*V*_*LA*_	Left atrium volume	28.9575	mL
*V*_*PV*_	Pulmonary vein volume	79.9216	mL
*V*_*LV*_	Left ventricle volume	49.7366	mL
*V*_*SA*_	Systemic artery volume	114.629	mL
*V*_*SV*_	Systemic vein volume	1427.75	mL
*V*_*RA*_	Right atrium volume	29.8093	mL
*V*_*RV*_	Right ventricle volume	37.8823	mL
*V*_*PA*_	Pulmonary artery volume	73.3862	mL
*R*_*AO*_	Impedance of systemic aorta	0.007	kPa s/mL
*R*_*SA*_	Systemic artery and capillary resistance	0.247	kPa s/mL
*R*_*SV*_	Systemic vein resistance	0.0514	kPa s/mL
*R*_*PV*_	Pulmonic vein resistance	5.05e−3	kPa s/mL
*R*_*AP*_	Pulmonic artery and capillary resistance	5.05e−3	kPa s/mL
*R*_*PA*_	Impedance of pulmonary artery resistance	0.004	kPa s/mL
*R*_*Mitral*_	Mitral valve resistance	5e−4	kPa s/mL
*R*_*Tricus*_	Tricuspid valve resistance	5e−4	kPa s/mL
*C*_*AS*_	Systemic arteries compliance	32.6	mL/kPa
*C*_*VS*_	Systemic vein compliance	433	mL/kPa
*C*_*VP*_	Pulmonic vein compliance	50.0	mL/kPa
*C*_*AP*_	Pulmonic artery compliance	41.7	mL/kPa
*E*_*LA*_ *Max.*	Maximum elastance of LA	0.0782	kPa/mL
*E*_*LA*_ *Min.*	Minimum elastance of LA	0.0711	kPa/mL
*E*_*RA*_ *Max.*	Maximum elastance of RA	0.03	kPa/mL
*E*_*RA*_ *Min.*	Minimum elastance of RA	0.0273	kPa/mL

### Model of IABP function

**Fig. 2:**
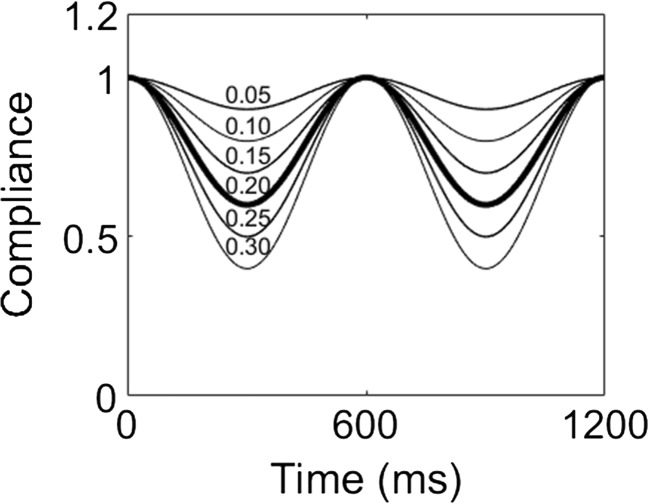
The variation in aortic compliances according to the level of balloon inflation, which is classified with various scale factor (*sf*) ranging from 0.05 to 0.3 in increments of 0.05. The compliance profile of the thick line, with a *sf* corresponding to 0.2, was selected for this computation simulation

Given the inflation and deflation of an IABP balloon inside a systemic artery, the cycle of its inflation and deflation was modeled as a time-varying compliance with respect to the systemic artery. A harmonic waveform was used for the time-varying compliance of the systemic artery to generalize the patterns of inflation and deflation of an IABP. The harmonic waveforms for the compliance of the artery were expressed as follows:

9 $$ {C}_{SA, IABP}(t)={C}_{SA}\times {SF}_{IABP}(t) $$

10 $$ {SF}_{IABP}(t)=\left(1- sf\right)+ sf\times \cos \left(\frac{2\pi t}{BCL}-\varnothing \right) $$

where *C*_*SA,IABP*_ denotes the time-varying compliance of the systemic arteries with the IABP, *C*_*SA*_ denotes the compliance of the systemic arteries without the IABP, *SF*_*IABP*_ denotes a scale factor for the IABP, *sf* denotes the level of the scale factor, and *BCL* denotes the cycle length of the ventricle and the time shift between the ventricular contraction cycle and the IABP inflation cycle. The *sf* represents a major parameter, which is proportional to the stroke volume of IABP and related to the pumping compliance of IABP. First, several *sf* parameter values such as 0.05, 0.1, 0.15, 0.2, 0.25, and 0.30 were applied to obtain appropriate model parameters of the IABP function (see Fig. 2). The *sf* parameter was selected as 0.2, and this reduced the *C*_*SA,IABP*_ value to 60% of the maximum value (Fig. 2). The pumping phase was set as 3.66 rad (350 ms shifted from the end-diastole), instead of the end-systole because the phase exhibited the maximum efficiency in terms of the volume of pumping blood during inflation as indicated by a previous study [5].

### Model of valve regurgitation

Two branches were added to both the aortic and the mitral compartments in the lumped parameter model to model MR and AR (Fig. 1). One branch has a forward diode to represent forward flow, and the other has a backward diode to represent leakage flow. The diodes have different resistance values. The regurgitant flow dynamics through the mitral and aortic valves are represented by the following equations:

11 $$ {Q}_{MI}=\left\{\begin{array}{c}\frac{P_{LA}-{P}_{LV}}{R_{MI}}\kern7.75em \mathrm{when}\ {P}_{LA}>{P}_{LV}\\ {}\frac{P_{LA}-{P}_{LV}}{R_{MI, Leak}}=\frac{P_{LA}-{P}_{LV}}{R_{MI}}\times \frac{SF}{100}\kern4.5em \mathrm{when}\ {P}_{LA}\le {P}_{LV}\end{array}\ \right. $$

12 $$ {Q}_{AO}=\left\{\begin{array}{c}\frac{P_{LV}-{P}_{AO}}{R_{AO}}\kern7.75em \mathrm{when}\ {P}_{LV}>{P}_{AO}\\ {}\frac{P_{LV}-{P}_{AO}}{R_{AO, Leak}}=\frac{P_{LV}-{P}_{AO}}{R_{AO}}\times \frac{SF}{100}\kern4.25em \mathrm{when}\ {P}_{LV}\le {P}_{AO}\end{array}\ \right. $$

where *Q*, *P*, and *R* denote the flow rate (mL/min), pressure (mmHg), and flow resistance (mmHg min/mL), respectively, and the subscripts *MI*, *AO*, *LV*, *LA*, and *Leak* represent mitral valve, aortic valve, left ventricle (LV), left atrium, and leakage, respectively. A scale factor for leakage blood flow through the valves was introduced to quantify the severity of the regurgitation. Several SF parameters, specifically 2, 4, 6, 8, and 10%, were applied to consider the variation in regurgitation fraction from weak to severe valvular insufficiency.

### Simulation protocol

For all simulations, the duration of the entire cardiac cycle was set as 600 ms. The severity of the regurgitation varied from 0% (baseline state) to 10% (severe regurgitation) in 2% increments. For each case, the simulation was executed for 20 s to ensure that the cardiovascular responses, such as blood pressure, flow, and volume, reached a nearly steady state in each compartment for a given degree of regurgitation. The changes in ventricular tension, contractile energy consumption, strain, stroke work, stroke volume, and regurgitant volume were computed for different values of regurgitation severity to analyze the effect of regurgitation on ventricular wall mechanics. The contractile energy consumption of the myocardium was quantified by calculating the contractile ATP consumption in the myofilament model proposed by Rice et al. [11]. The contractile ATP consumption rate, denoted by *E*, per unit volume was calculated as a function of the ATP-consuming cross-bridge detachment rate (*g*_*xbT*_ ) and the single overlap fraction of thick filaments (*SOVF*_*Thick*_), using the following equation:

13 $$ \mathrm{Contractile}\ \mathrm{ATP}\ \mathrm{consumption}\  \mathrm{rate}={g}_{xbT}\times {SOVF}_{Thick} $$

where *g*_*xbT*_ indicates the ATP-consuming detachment transition rate and *SOVF*_*Thick*_ indicates the single-overlap fraction of the thick filament; these functions were obtained from the original myofilament model proposed by Rice et al. [11].

## Results

**Fig. 3:**
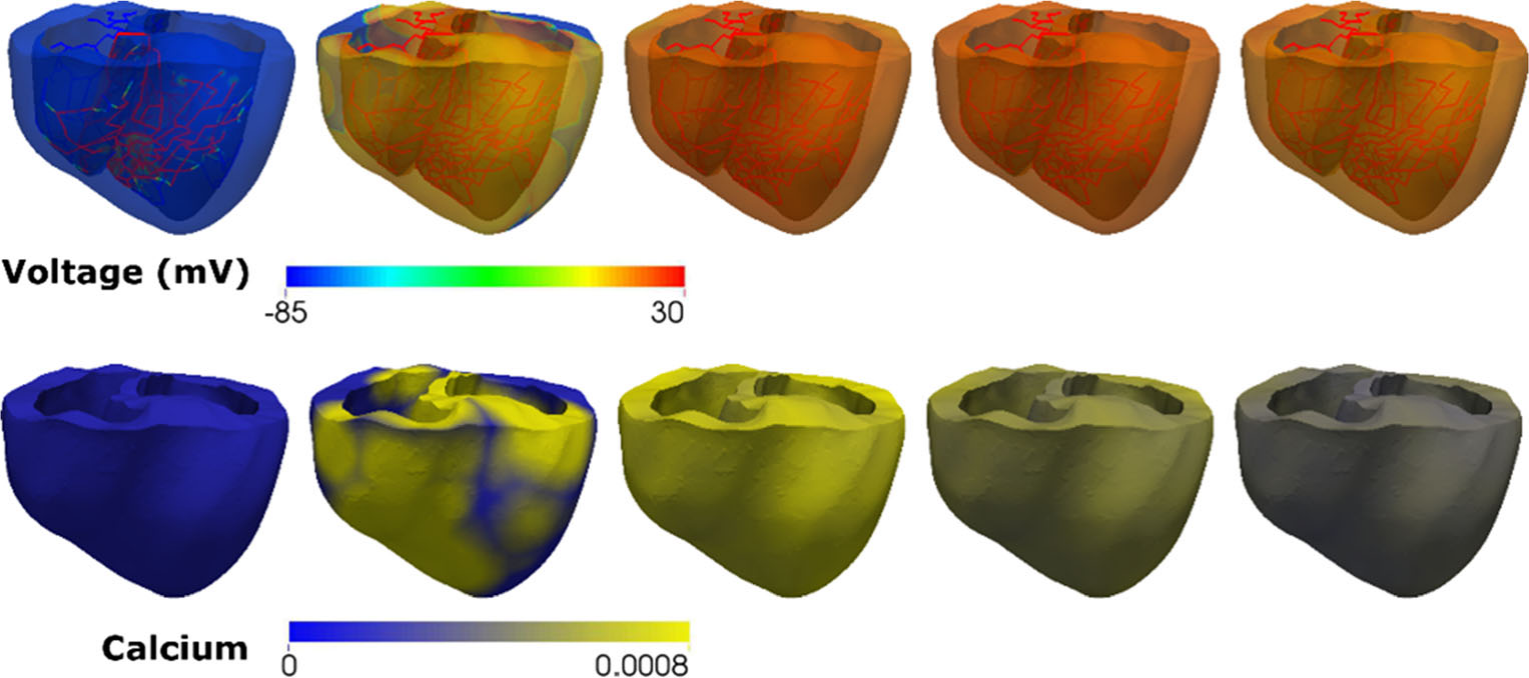
Transmural distribution of membrane potentials and Ca^2+^ concentration with respect to time in sinus rhythm induced through the Purkinje network pathway

Figure 3 shows the transmural distribution of membrane potential (Fig. 3a) and intracellular Ca^2+^ concentration (Fig. 3a) with respect to time in the sinus rhythm induced through the Purkinje network pathway. The intracellular Ca^2+^ concentrations throughout the ventricles were mapped onto the mechanical mesh of the ventricle as input parameters to trigger cross-bridge cycling for all the cases of mechanical simulation.

**Fig. 4:**
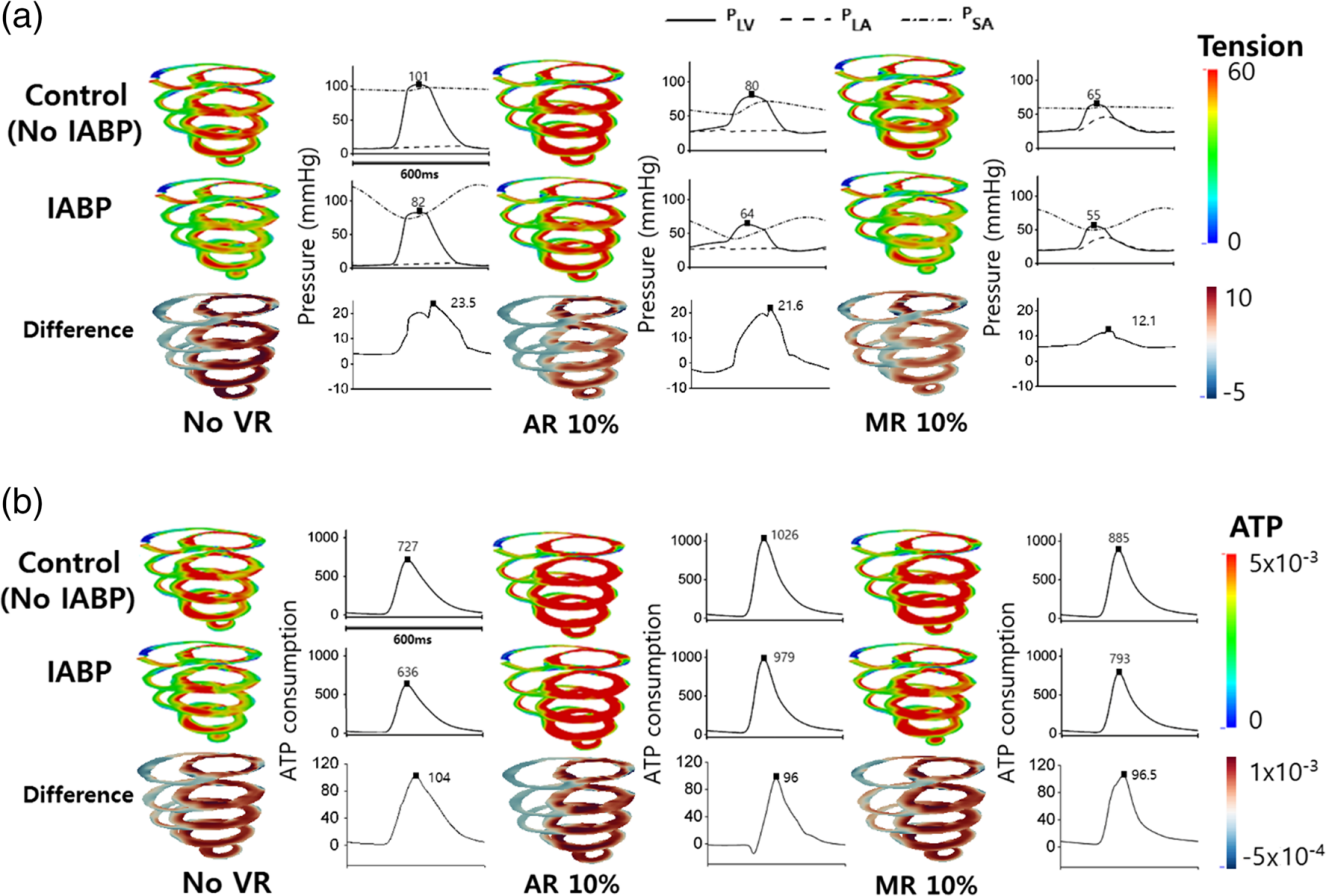
Transmural distribution of myocardial contractile ATP consumption rate and tension and blood pressure in the LV, aorta, and LA under 10% AR and 10% MR conditions in the control and IABP therapy groups

Figure 4 shows the transmural distribution of myocardial tension and contractile ATP consumption rate, and blood pressure in the LV, aorta, and LA under 10% AR and 10% MR conditions across the control and IABP therapy groups. The differences in tension and ATP consumption were computed by subtracting tension and ATP consumption in the IABP group from their respective values in the control group, which represents the group not treated by IABP. According to Fig. 4a, myocardial tension is higher in AR, while the generated LV pressure is lower in AR when compared with that in the no VR condition. The LV pressure was the lowest in the MR condition. In the IABP therapy group, the ventricles developed less myocardial tension and intraventricular pressure in all the cases when compared with the control group. The maximum reduction in myocardial tension due to the IABP was obtained in the no VR condition. The reduction in myocardial tension decreased according to the severity of the VR. Moreover, the reduction in myocardial tension was more significant in the MR when compared with that in the AR condition. The VR resulted in increasing the ATP consumption of the ventricles with respect to the ventricular contraction. The contractile myocardial ATP consumption was the highest in the AR condition (Fig. 4b). Contractile myocardial ATP consumption increased according to the severity of VR and was higher in the AR condition when compared with that in the MR condition. Although the contractile ATP consumption was reduced by IABP therapy in all three cases, the highest consumption was obtained in the AR condition. The reduction ratio of contractile ATP consumption was the highest (13% reduction) in the no VR condition, 10% in the MR condition, and 5% in the AR condition. According to these results, IABP therapy was disturbed in terms of the reduction in myocardial tension generation and contractile ATP consumption due to valvular regurgitation, particularly in the AR condition.

**Fig. 5:**
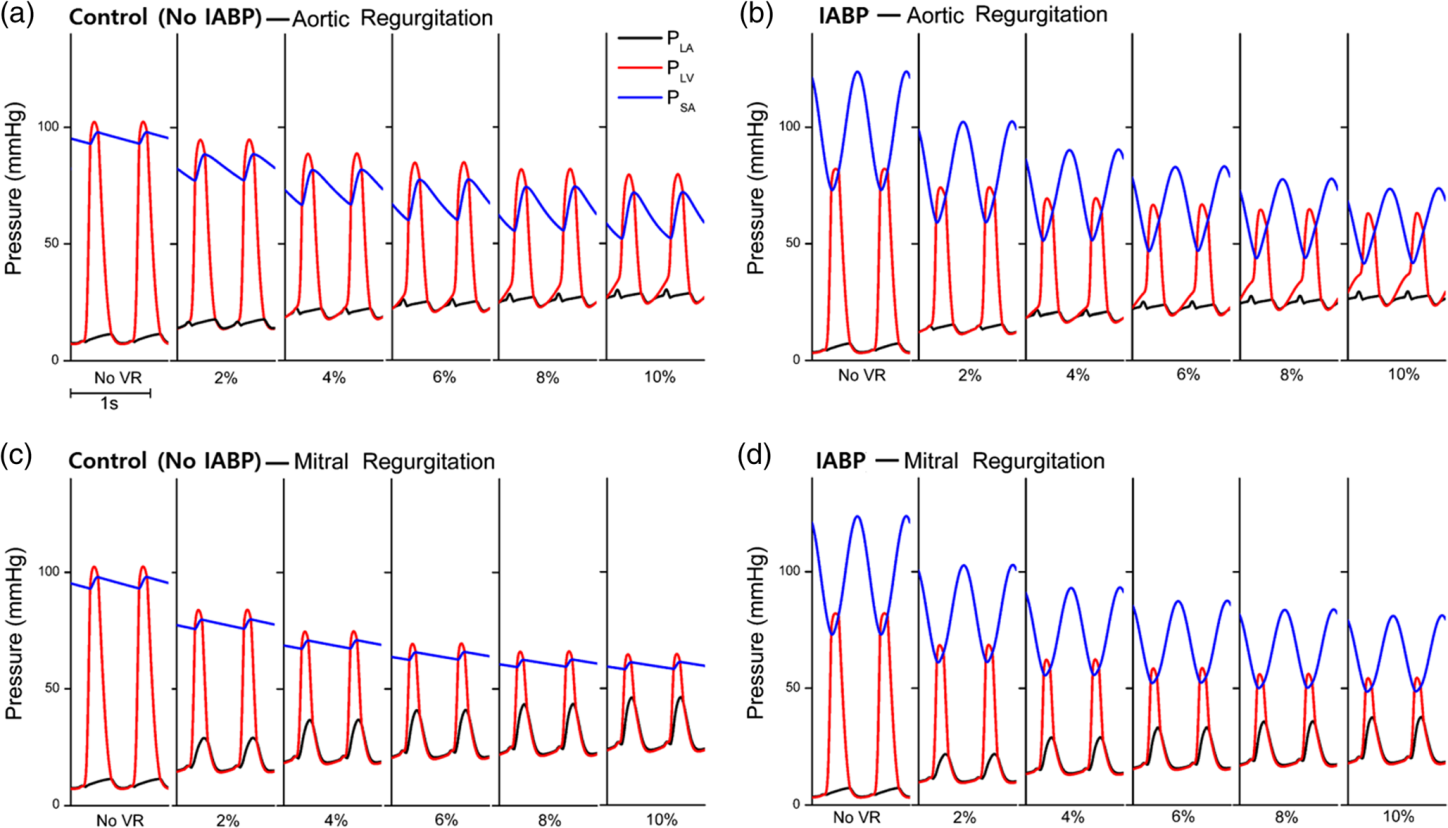
Pressure profiles of the left atrium, left ventricle, and systemic arteries under the condition of no valve regurgitation and varying severities of aortic regurgitation and mitral regurgitation in the no IABP group and IABP group

Figure 5 shows the pressure profiles of LA, LV, and the systemic arteries under the condition of no VR. Moreover, it shows the varying severities of AR and MR in the control and IABP groups. Additionally, the LV peak pressure and arterial pressures decreased according to the severity of the VR (Fig. 5a, c). However, arterial pulse pressure increased according to the severity of AR. The IABP reduced the LV systolic pressures and increased the arterial pressure in the diastole period owing to the counterpulsation mechanisms in both the AR and MR conditions (Fig. 3b, d). The counterpulsation effect due to the IABP (Fig. 3b, d) corresponds to previous experimental studies [16–18]. The atrial pressures under the VR conditions were higher than that under the no VR condition. In particular, under the MR condition, the left atrial pressure shape was similar to the LV pressure profile, whereas the pressure shape did not follow the shape of the LV pressure profile in the AR condition. Although the IABP decreased the enhancement of left atrial pressure slightly in the AR condition, it did not significantly help in reducing the pressure to its normal value.

**Fig. 6:**
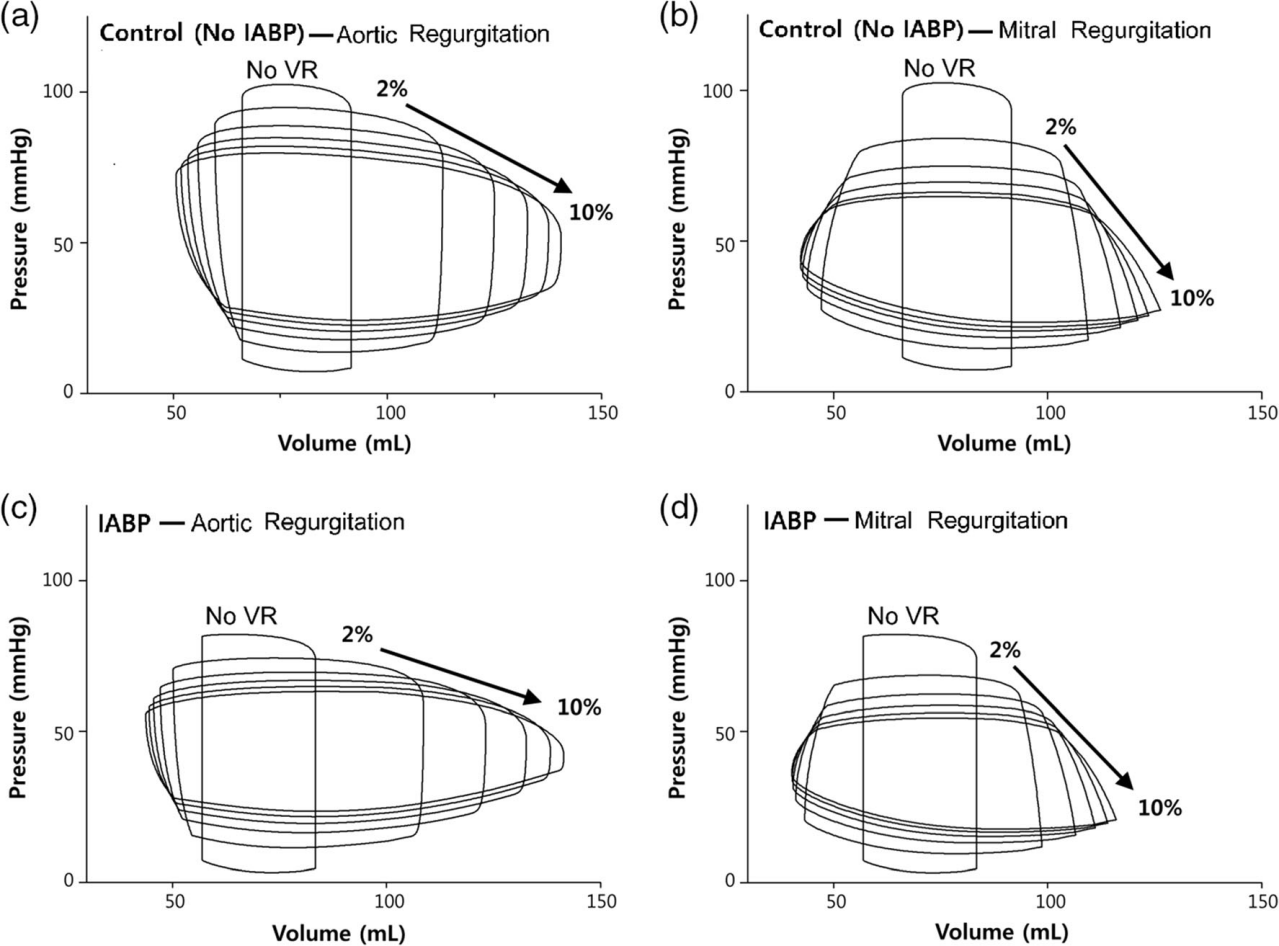
Pressure–volume loops according to the severity of the AR and MR in the control and IABP therapy groups

Figure 6 shows the pressure–volume loops for different degrees of AR and MR in the control and IABP therapy groups. The difference between systolic and diastolic pressure decreased as the VR severities increased (Fig. 4a). Furthermore, the end-diastolic volume (EDV) increased and end-systolic volume (ESV) decreased, thereby forming a horizontally extended pressure–volume loop in the VR condition. When the IABP was treated, there was a further increase in the EDV and a further decrease in the ESV in the AR condition, thereby forming a more horizontally extended pressure–volume loop. However, the EDV decreased and ESV remained constant in the MR condition, thereby forming a shape of the loop that was similar to the shape without the IABP treatment. These results indicated that the IABP worsened the problem of ventricular expansion induced due to regurgitated blood volume during the diastole under the AR condition.

Regurgitant volume and fraction increased sharply at low levels of regurgitant severity, but the increase was proportional to the increased severities of the AR and MR (Fig. 4a, b). The IABP further increased regurgitated blood volume at all the severity levels of the AR, but decreased regurgitated volume at all the severity levels of the MR. Accordingly, the IABP treatment under the AR condition resulted in more serious levels of blood regurgitation through the aortic valve.

Generally, stroke volume is calculated by subtracting ESV from EDV in the pressure–volume loop. However, practical SV was not equal to EDV–ESV under the regurgitation conditions. The EDV–ESV refers to the degree of ventricular movement as opposed to the ventricular stroke volume. Therefore, practical SV was computed by using arterial blood flow during a single cycle. The SV and the ejection fraction calculated according to this method decreased in both the AR and MR with increased regurgitant volume (Fig. 5c, d). The SV and EF increased slightly in the AR condition, but decreased slightly in the MR condition when the IABP was treated. In terms of the cardiac output, IABP therapy under the MR condition disturbed ventricular pumping.

Stroke work is defined as the amount of work performed by the ventricle to pump blood out of ventricle. It can be estimated in a manner similar to the area within the pressure–volume loop. Interestingly, as the regurgitant volume increased, the stroke work increased for weak regurgitation severity conditions, but subsequently decreased for severe regurgitation conditions (Fig. 5e). The LV stroke work decreased at all the severities of regurgitation when the IABP was treated. Accordingly, the IABP reduced ventricular working load even under the VR condition.

Contractile ATP consumption refers to the amount of ATP molecules used for cross-bridge cycling. It was higher under larger regurgitant volumes in the AR and MR conditions. However, the IABP reduced contractile ATP consumption in all the cases in the AR and MR conditions. Accordingly, the IABP retrenched biological energy in pumping blood.

## Discussion

**Fig. 7:**
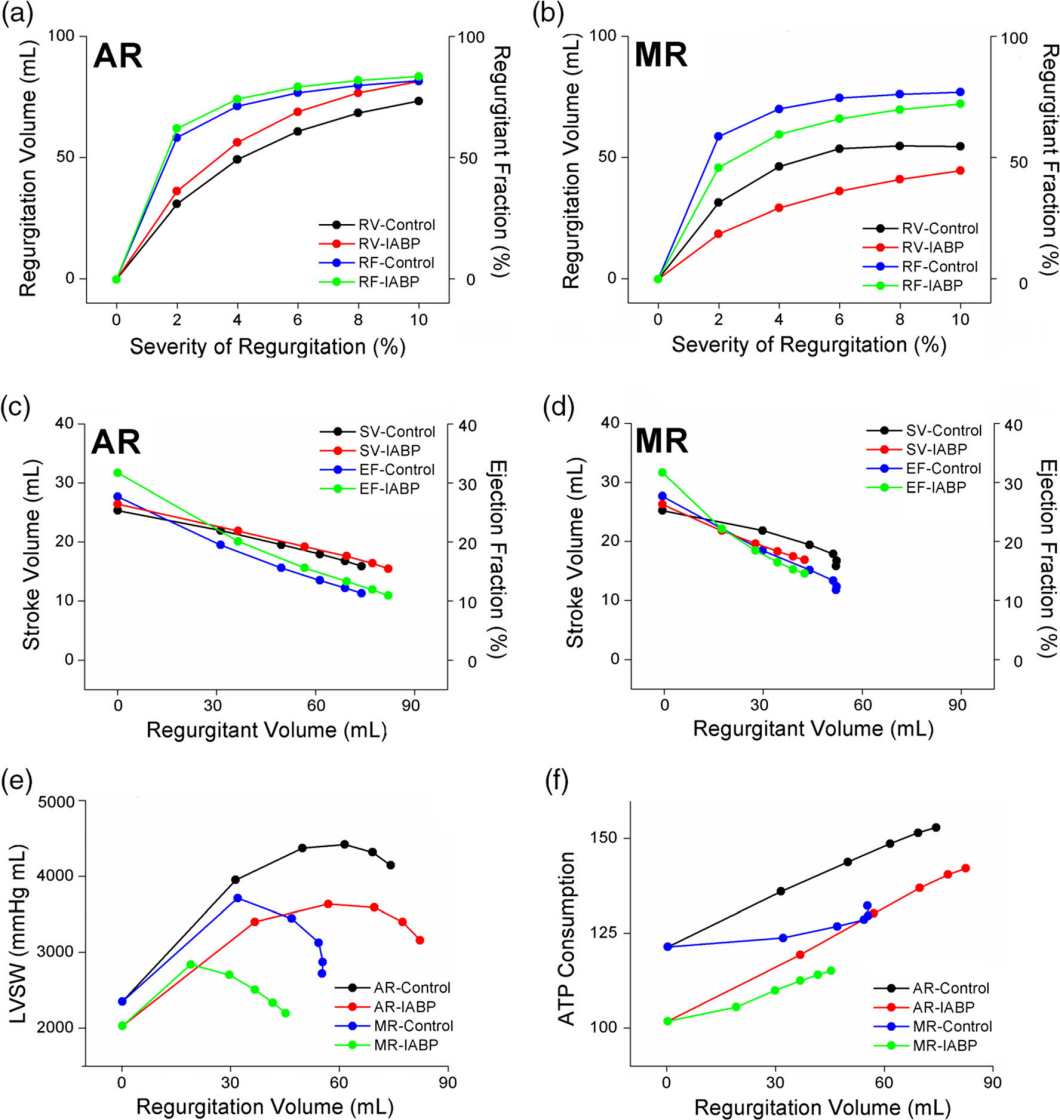
Regurgitant volumes and fractions vs. severity of regurgitation (**a**, **b**), stroke volume, and ejection fraction vs. regurgitant volume (**c**, **d**), in the AR and MR. Left ventricular stroke work (**e**) and ATP consumption rate (**f**) according to the severity of the AR and MR in the control and IABP therapy groups. In the figure, LV denotes the left ventricle, RV denotes the right ventricle, SV denotes the stroke volume, LVSW denotes the left ventricular stroke work, ATP denotes the contractile ATP consumption rate, and SV denotes the stroke volume

In this study, the effect of IABP on heart failure with valvular regurgitation (AR and MR) was quantitatively examined by using a sophisticated computational model of IABP-implanted ventricles. The main findings were follows:

In this study, the effect of IABP on heart failure with valvular regurgitation (AR and MR) was quantitatively examined by using a sophisticated computational model of IABP-implanted ventricles. The main findings are as follows:

1. IABP therapy was disturbed in terms of reducing the myocardial tension generation and contractile ATP consumption by valvular regurgitation, particularly in the AR condition (Fig. 4).
2. The IABP worsened the problem of ventricular expansion induced owing to the regurgitated blood volume during the diastole under the AR condition (Fig. 6).
3. The stroke volume and ejection fraction (which are important indices for estimating cardiac pumping function) decreased with increasing severity of valvular regurgitation. The IABP enabled an increase in stroke volume in the AR condition, but reduced the stroke volume further in a severe MR condition (Fig. 7c, d).
4. Contractile myocardial ATP consumption increased with the severity of the AR and MR, whereas the IABP reduced the contractile myocardial ATP consumption (Fig. 6c). The IABP retrenched biological energy in pumping blood in the AR and MR conditions, as well as in the no regurgitation condition. In addition, the IABP reduced the LV stroke work under the AR, MR, and no regurgitation conditions (Fig. 7e).

The electromechanical model presented in this study represents a new approach for understanding the effects of valvular insufficiency and the benefit of IABP, particularly with respect to hemodynamics, myocardial tension, and contractile energy consumption. The reduction of myocardial tension and contractile ATP consumption by IABP were significantly reduced in the AR condition (Fig. 4). Therefore, the IABP was disturbed, resulting in a reduction in myocardial tension generation and contractile ATP consumption, which are supposed to represent the role of IABP therapy in terms of ventricular unloading.

According to Fig. 5, the left atrial pressure increased significantly when compared with that in the no VR condition. The blood regurgitated into the left atrium when the ventricles contracted in the MR condition. This increase in the left atrial pressure in the MR condition could induce pulmonary edema.

Generally, the sarcomere length affects the density of effective cross-bridges and therefore influences the contractile force. This is known as the Frank–Starling law of the heart. Therefore, a longer sarcomere length results in greater myocardial tension and contractile ATP consumption due to the greater density of the attached cross-bridges in the myocytes. The blood could easily regurgitate into the LV due to the aortic valve insufficiency when the IABP is inflated, but the blood only flowed forward to systemically circulate in the MR condition. Accordingly, the EDV was decreased in the MR condition due to the IABP treatment, although it was even increased when treated by the IABP in the AR condition (Fig. 6). This was the reason for the increased reduction in the myocardial tension and contractile ATP consumption in the MR condition when compared with that in the AR condition. If this AR condition persists in the long term, then it could lead to dilated cardiomyopathy.

The present study has several limitations. Although a 3D multi-scale model of ventricles was used, the vascular system and atria were simply modeled as lumped parameter models based on a Windkessel element. Additionally, the dynamics of aortic and mitral valves were not mimicked but were considered as static one-way check valves to reduce the model complexity. Furthermore, the dynamics of the IABP device were not considered realistically, but the function of inflation and deflation of the IABP with the time-varying compliance of the aorta was applied. Only the contractile ATP consumption of the myocardium was considered, although the ATP was used in other ways such as sarcoplasmic/endoplasmic reticulum calcium ATPase (SERCA), plasma membrane Ca^2+^-ATPase (PMCA), and Na pumps. Moreover, the coronary circulation was not implemented to reduce modeling complexity. However, these limitations are not expected to significantly alter the main findings of this study.

The computational model of cardiac electromechanics proposed in this study was used to quantitatively predict the effect of IABP function on the cardiovascular responses of patients with AR and MR conditions. The IABP could partially perform its role in the MR condition. However, it was disturbed by the AR and worsened the cardiovascular responses that followed the AR. Therefore, this study computationally proved the reason for the clinical contraindication of IABP in AR patients.

## References

[1] Staier K, Wilhelm M, Wiesenack C, Thoma M, Keyl C (2012). Pulmonary artery vs. transpulmonary thermodilution for the assessment of cardiac output in mitral regurgitation: a prospective observational study. Eur J Anaesthesiol.

[2] Dekker AL, Reesink KD, van der Veen FH, van Ommen GV, Geskes GG, Soemers AC, Maessen JG (2003). Intra-aortic balloon pumping in acute mitral regurgitation reduces aortic impedance and regurgitant fraction. Shock.

[3] Levine AJ, Dimitri WR, Bonser RS (1999). Aortic regurgitation in rheumatoid arthritis necessitating aortic valve replacement. Eur J Cardiothorac Surg: Off J Eur Assoc Cardiothorac Surg.

[4] Lim KM, Constantino J, Gurev V, Zhu R, Shim EB, Trayanova NA (2012). Comparison of the effects of continuous and pulsatile left ventricular-assist devices on ventricular unloading using a cardiac electromechanics model. J Physiol Sci: JPS.

[5] Lim KM, Lee JS, Gyeong MS, Choi JS, Choi SW, Shim EB (2013). Computational quantification of the cardiac energy consumption during intra-aortic balloon pumping using a cardiac electromechanics model. J Korean Med Sci.

[6] Lim KM, Hong SB, Lee BK, Shim EB, Trayanova N (2015). Computational analysis of the effect of valvular regurgitation on ventricular mechanics using a 3D electromechanics model. J Physiol Sci: JPS.

[7] Lim KM, Jeon JW, Gyeong MS, Hong SB, Ko BH, Bae SK, Shin KS, Shim EB (2013). Patient-specific identification of optimal ubiquitous electrocardiogram (U-ECG) placement using a three-dimensional model of cardiac electrophysiology. IEEE Trans Biomed Eng.

[8] Gurev V, Lee T, Constantino J, Arevalo H, Trayanova NA (2011). Models of cardiac electromechanics based on individual hearts imaging data: image-based electromechanical models of the heart. Biomech Model Mechanobiol.

[9] Berenfeld O, Jalife J (1998). Purkinje-muscle reentry as a mechanism of polymorphic ventricular arrhythmias in a 3-dimensional model of the ventricles. Circ Res.

[10] ten Tusscher KH, Panfilov AV (2006). Alternans and spiral breakup in a human ventricular tissue model. Am J Phys Heart Circ Phys.

[11] Rice JJ, Wang F, Bers DM, de Tombe PP (2008). Approximate model of cooperative activation and crossbridge cycling in cardiac muscle using ordinary differential equations. Biophys J.

[12] Kerckhoffs RC, Neal ML, Gu Q, Bassingthwaighte JB, Omens JH, McCulloch AD (2007). Coupling of a 3D finite element model of cardiac ventricular mechanics to lumped systems models of the systemic and pulmonic circulation. Ann Biomed Eng.

[13] Helm RH, Byrne M, Helm PA, Daya SK, Osman NF, Tunin R, Halperin HR, Berger RD, Kass DA, Lardo AC (2007). Three-dimensional mapping of optimal left ventricular pacing site for cardiac resynchronization. Circulation.

[14] Wu Y, Bell SP, Trombitas K, Witt CC, Labeit S, LeWinter MM, Granzier H (2002). Changes in titin isoform expression in pacing-induced cardiac failure give rise to increased passive muscle stiffness. Circulation.

[15] O’Rourke B, Kass DA, Tomaselli GF, Kaab S, Tunin R, Marban E (1999). Mechanisms of altered excitation-contraction coupling in canine tachycardia-induced heart failure, I: experimental studies. Circ Res.

[16] Krishna M, Zacharowski K (2009). Principles of intra-aortic balloon pump counterpulsation. Contin Educ Anaesth Crit Care Pain.

[17] Kolyva C, Pantalos GM, Giridharan GA, Pepper JR, Khir AW (2009). Discerning aortic waves during intra-aortic balloon pumping and their relation to benefits of counterpulsation in humans. J Appl Physiol.

[18] Parissis H, Graham V, Lampridis S, Lau M, Hooks G, Mhandu PC (2016). IABP: history-evolution-pathophysiology-indications: what we need to know. J Cardiothorac Surg.

